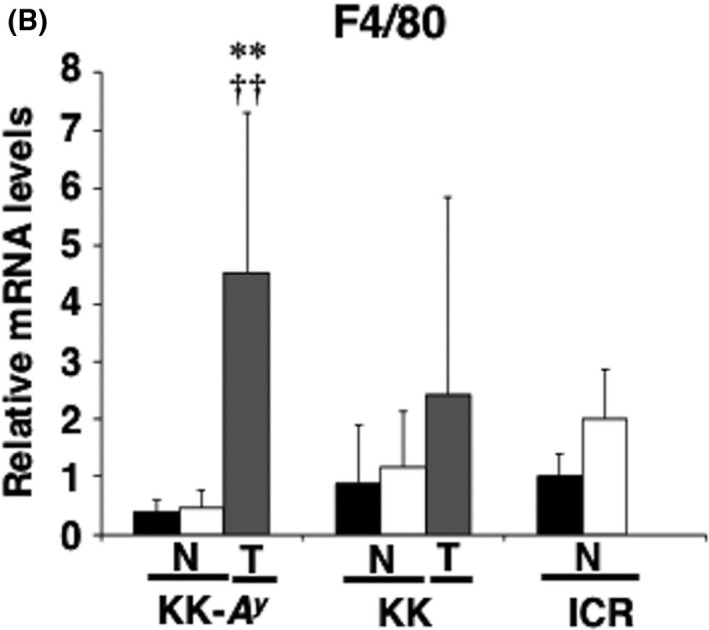# Correction

**DOI:** 10.1111/cas.14417

**Published:** 2020-05-15

In an article^1^ titled “A^y^ allele promotes azoxymethane‐induced colorectal carcinogenesis by macrophage migration in hyperlipidemic/diabetic KK mice” by Kumiko Ito, Rikako Ishigamori, Michihiro Mutoh, Toshihiro Ohta, Toshio Imai, Mami Takahashi, Figure 7b is to be corrected.

The authors apologize for the error.

1

**FIGURE 7:**